# Structure of the Red Blood Corpuscles

**Published:** 1872-02

**Authors:** 


### SELECTED ARTICLES.

### ARTICLE VII.

### *Structure of the Red Blood-Corpuscles.*

Nothing can better illustrate the difficulties that beset the determination of the minute points of microscopical inquiry than the discrepancy of opinion that exists amongst the best observers in regard to the structure of the red blood-corpuscle. For many years it was held to be indisputably a cell, and to consist of a definite cell-wall enclosing cell-contents. For sometime past, however, a change of opinion has been visible; and in most of our text-books of physiology, if it be not expressly stated, it is at least hinted at as probable, that the corpuscles are homogeneous semi-solid bodies, the surface of which may perhaps be a little more condensed than the interior. The remarkable experiments of Mr. Roberts, of Manchester, on the action of the anilin and tannin, though at first apparently in favor of the cell theory, were yet subsequently considered to be explicable on the theory of homogeneity, by supposing that these agents hardened the surface, and so led to the phenomena observed. The peculiarity and persistence of the form of the red corpuscles, and their behavior on the application of pressure, are certainly in favor of this latter view. A paper, however, by Dr. Joseph Richardson, of Philadelphia, which we have just received, speaks strongly in favor of the old cellular view. This gentleman's experiments were conducted upon the Menobranchus, which he obtained from the Cayuga lake in Western New York, the blood corpuscles of which animal are, as is well known, gigantic, being about 216 times larger than those of man. In endeavoring to discover some indications of the presence of a cell-wall, he found quite unexpectedly that the colored portion possesses the remarkable property of crystalising with great readiness *within* its envelope. Dr. Richardson states that, on slightly concentrating the blood of this animal, one or two crystals form in almost every corpuscle; and the effect of their formation and elongation is precisely what we might expect to be produced by bodies of similar shape contained within an ordinary bladder partially filled with fluid, the ends of the corpuscle being in some instances thrust out till the length becomes a third greater and its breadth correspondingly diminished, the nucleus being closely compressed against the prism. In other instances, where the corpuscles lie across, the whole corpuscle assumes a lozenge or rectangular form, in which state it may be mounted dry. Dr. Richardson further argues—though this is less satisfactory evidence—that on briskly stirring, freshly drawn blood with several times its volume of water, the coloring matter can be withdrawn, leaving the cell membrane intact. And finally, he has succeded in dividing a corpuscle under the microscope with a sharp needle; the contents escaped, while the cell-wall shrank up around the nucleus into a perfectly hyaline particle. From these researches he concludes that the older theory, which asserts that the red corpuscle of the vertebrates generally are vesicles, each composed of a delicate, colorless, inelastic, porous, and perfectly flexible cell-wall, enclosing a colored fluid, which is sometimes crystallizable and is freely miscible with water, explains the physical phenomena presented by the red globule far more satisfactorily than any other hypothesis that has hitherto been advanced.

Without disputing the accuracy of the observations here recorded in reference to the corpuscles of the Amphibia we would just remark that it by no means follows that the structure of the corpuscles of the higher animals is at all similar; and we are still disposed to hold the opinion of Dr. Gulliver, that, in mammals at least, the red corpuscles are nuclei, and as such are probably homogeneous in composition, and destitute at any rate of a proper cell-wall.—*London Lancet.*

---

